# Questioning current definitions for breastfeeding research

**DOI:** 10.1186/1746-4358-7-9

**Published:** 2012-08-13

**Authors:** Joy Noel-Weiss, Sonya Boersma, Sonya Kujawa-Myles

**Affiliations:** 1 School of Nursing, University of Ottawa, 451 Smyth RGN3051, Ottawa, ON, K1H 8M5, Canada

**Keywords:** Breastfeeding, Definitions, Duration, Exclusivity, Breast milk, Relationships, Pumping, Expressing

## Abstract

**Background:**

The aim of this paper is to examine how breastfeeding is defined for research purposes.

**Discussion:**

Current breastfeeding definitions focus on the amount of breast milk an infant receives and do not encompass how a baby is fed. Our concerns are that key variables are not measured when mothers are pumping or expressing their milk and bottle feeding. It seems the breastfeeding relationship is not considered in the definition.

**Conclusion:**

While we appreciate the implications of full versus partial breastfeeding in research studies, we also believe the method of infant feeding to be significant. Researchers should develop new definitions.

## Introduction

Which baby is exclusively breastfed? One baby, aged three months, is always fed at his mother's breast (note that we refer to infants in the masculine, mothers are feminine). He has never had a bottle. About one quarter of his feedings are supplemented with infant formula provided through a small tube at his mother's breast. A second baby, also three months old, has received only breast milk from birth. All breast milk has been provided by bottles fed to him by numerous people. About half of the breast milk has been donated by other women. According to the current definitions of breastfeeding, the second baby is exclusively breastfed because the amount of breast milk determines breastfeeding status while the method of feeding is not considered in the definition [1,2]. Table 1 provides the WHO definitions for infant feeding and Table 2 provides the definitions according to the Interagency Group for Action on Breastfeeding [1,2]. 

**Table 1:** WHO definitions for infant feeding (WHO, 2008)

**Feeding category**	**Infant receives**	**May include**	**Does not include**
Exclusive breastfeeding	Breast milk (including milk expressed or from a wet nurse)	ORS, drops, syrups (vitamins, minerals, medicines)	Anything else
Predominant breastfeeding	Breast milk (including milk expressed or from a wet nurse) as the predominant source of nourishment	Certain liquids (water and water-based drinks, fruit juice), ritual fluids and ORS, drops or syrups (vitamins, minerals, medicines)	Anything else (in particular, non-human milk, food-based fluids)
Complementary feeding	Breast milk (including milk expressed or from a wet nurse) and solid or semi-solid foods	Anything else: any food or liquid including non-human milk and formula	NA
Breastfeeding	Breast milk (including milk expressed or from a wet nurse) and solid or semi-solid foods	Anything else: any food or liquid including non-human milk and formula	NA
Bottle-feeding	Any liquid (including breast milk) or semi-solid food from a bottle with nipple/teat	Anything else: any food or liquid including non-human milk and formula	NA

**Table 2:** Interagency group for action on breastfeeding (Labbok & Krasovec, 1990)

**First level**	**Second level**	**Infant receives**
Full breastfeeding	Exclusive	no other liquid or solid is given to infant
	Almost exclusive	vitamins, minerals, water, juice, or ritualistic feeds given frequently in addition to breastfeeds
Partial breastfeeding	High	more than 80 % breastfeeds
	Medium	between 20 and 80 %
	Low	less than 20 %
		minimal, occasional, irregular breastfeeds

Clinicians, statisticians, policymakers, and researchers need accurate and consistent definitions for breastfeeding. What and how an infant is fed is relevant to a clinician assessing infant growth and development or ordering a medication for his mother. Statisticians and policymakers use breastfeeding indicators to establish infant feeding trends and to decide future policies [1]. Breastfeeding and lactation researchers need definitions that accurately describe breastfeeding and allow comparisons of research studies.

The purpose of this paper is to question the current definitions for breastfeeding. In particular, the aim is to examine how breastfeeding is defined for research purposes. The essence of this analysis is to question the way definitions are based on *what* the baby is fed to the exclusion of *how* a baby is fed.

## Background

Duration and exclusivity are the key measures of breastfeeding patterns [2-5]. Duration is the length of time for any breastfeeding, including breastfeeding through the initial stage of exclusive breastfeeding and any period of complementary feeding until weaning [3,6]. Exclusivity is a measure of the amount of breastfeeding without supplementation (e.g., infant formula or other breast milk replacements), and 6 months of age is a key marker since complementary foods (i.e., solids) usually begin around 6 months postpartum [4,5]. Supplementation, in this case, is a breast milk replacement, whereas complementary foods are provided in addition to breast milk when the child is developmentally ready for solid food [7].

Researchers need precise and reliable definitions for breastfeeding. If breastfeeding is the independent variable and outcomes are correlated or attributed to it, then researchers need to establish that babies were actually fed breast milk. For this reason, the current definitions were built to differentiate between exclusive and partial breastfeeding [2].

Labbok and Krasovec report the work of an interagency group that developed the original work about infant feeding categories [2]. They provided the terms and definitions that form the basis for categories used by many breastfeeding and lactation researchers [3,8-11]. The World Health Organization has set a standard for definitions used primarily for population monitoring by asking about the last 24 hours [1]. Aarts et al. recognized that the term "exclusive breastfeeding" can be misleading when woman are asked for current status of feeding versus how baby has been fed since birth [12]. Their conclusion was that amount of breastfeeding should be measured from birth if an infant is called "exclusively breastfed" [12]. Thulier proposed a new, shorter list for definitions, stating that current definitions lack clarity and emphasizing that definitions must be based on content, not mode of feeding [13]. The common thread is that food, not feeding method, forms the definition for breastfeeding.

## Discussion

### Patterns

Breastfeeding patterns can vary over the first six months postpartum, and this fact makes definitions for breastfeeding a challenge [14]. Supplements of infant formula may be given in hospital and never again. Breastfeeding could be stopped due to illness or other factors, but then full breastfeeding could resume afterwards. Mothers might go away for a weekend and then resume normal breastfeeding routines after returning. Some mothers experiment with solids, and then discontinue them until their babies are older. It is difficult to capture a quantifiable pattern.

For the most part, the algorithms researchers use ask mothers how their babies were fed at a point in time, not over time. The research question usually determines the time points for tracking infant feeding patterns. For example, a researcher might track how babies are fed at two months, four months, and six months. At each of these time points, the mother might be asked to recall the past 24 hours or past seven days. Such methods and questions are assumed to inflate the amount of exclusive breastfeeding since the use of infant formula could have occurred in the non-recorded times [1,12,15].

### Product

Naturally, what babies are fed is important to researchers. For example, if a researcher is looking at the effects of, or correlations between, breastfeeding and obesity or between breastfeeding and illnesses, such as diabetes or ear infections, then how much breast milk is consumed and how long breast milk intake continues are important measurements.

Breastfeeding can take many forms and patterns, and it may include supplements of infant formula or solid food. It appears the benefits of breastfeeding are dose related and both exclusive and extended breastfeeding are optimal [16-19]. In the process of standardizing breastfeeding definitions, only the actual "dose" the infant was receiving was recognized. The current definitions separate breast milk fed (full dose) from partially breast milk fed (partial dose). In addition to exclusivity, researchers may track duration and how long the "dose" continued.

### Process

Pumping and bottling or expressing and feeding babies away from their mothers' breasts has become a common method for infant feeding [20]. Noel-Weiss et al., when researching maternal self-efficacy (i.e., a woman's confidence in her ability to breastfeed), realized that the current categories do not take into account *how* the baby is fed (i.e., feeding at mother's breast versus fed manually expressed or pumped breast milk; breastfed versus breast milk fed, respectively) [21,22]. This distinction is important when studying maternal self-efficacy, since feeding at mother's breast challenges a woman's confidence differently than feeding pumped and bottled milk [21].

Distinguishing between the product and the process may also enlighten researchers when the topic is not about self-efficacy. For example, the process of breastfeeding (e.g., option of non-nutritive sucking, consistent caregiver, skin-to-skin, and positioning) may be a mediating or moderating factor affecting the phenomenon under study (e.g., obesity, diabetes, or ear infections). While the product may be a key factor to preventing future obesity, maintaining an optimal adult weight might also be linked to babies having the option of non-nutritive sucking at their mothers' breasts. It is possible that the comfort of a mother's touch is lowering cortisol levels which might be a factor in diabetes. Perhaps the positioning of a nursing baby reduces the possibility of an ear infection. Researchers should include the mode of feeding when defining breastfeeding.

In the case of research, misinterpretations of the evidence are possible when how the baby is fed is not considered. For example, breast milk is known to have relevant immunological factors [17,18]. How the baby is fed might affect these properties in two ways; when the milk is altered and when the immune response is not triggered. Mothers produce antibodies to the viruses they are exposed to and their babies benefit from these antibodies [17,18]. If the milk is pumped and stored or pasteurized then the immune properties might be altered. Additionally, when the breast milk is provided by other women, then the antibodies are no longer tailored to a woman's own environment and her own baby. There is evidence that if an infant contracts a virus before his mother, the act of sucking will cause his mother's breasts to build antibodies [23]. If the act of suckling triggers an immune response, then lack of contact between the mother and baby (e.g., by pumping and bottling) nullifies this potential immune response.

The subheadings "product" and "process" used here were deliberate and are intended to be tongue-in-cheek. The current definitions reflect a reverence for breast milk and a disregard for the breastfeeding relationship. However, separating the milk from the mother and disregarding the relationship might miss key variables that contribute to health outcomes.

## Conclusion

In this commentary, we question several aspects of the current breastfeeding definitions. In particular, we question how the definitions are based on *what* the baby is fed to the exclusion of *how* a baby is fed. Fundamental to our concerns is the loss of the relationship. Breastfeeding benefits appear to be dose related, but one should consider if the dose ought to be restricted to the amount of milk. Perhaps the positive health outcome is also related to a "dose" of mothers' arms, distinctive sucking patterns, and nursing at mothers' breasts.

An accurate definition of breastfeeding will be complex. While the amount of breast milk taken is a key part of the definition, the patterns and mode of feeding should also be considered. Regarding exclusivity and patterns, two different definitions might need to be developed; one for a point-in-time measure and one for a life-long pattern. Determining the method of feeding should also be assessed, as it could be a significant mediator or moderator for health outcomes.

## Competing interests

The authors declare that they have no competing interests.

## Authors' contributions

JN-W conceived the commentary and wrote the first draft. SB and SK-M contributed to the subsequent drafts. All authors approved the final manuscript.

## Authors' information

JN-W RN IBCLC PhD is an assistant professor at the School of Nursing, University of Ottawa. JN-W is an experienced nurse and lactation consultant who has worked in hospital and community settings, and she is building a research program on the topic of breastfeeding and lactation.

SB RN BN IBCLC is a Masters of Nursing student at the School of Nursing, University of Ottawa. SB is an experienced nurse and lactation consultant in private practice, in various breastfeeding support drop-ins, in public health and is currently also engaged in lactation research. SB's Masters is focused on human lactation.

SK-M RN BScN IBCLC is a Masters of Nursing Science student at the University of Ottawa and is currently involved in breastfeeding research. SK-M has worked in a variety of hospital and community settings and is currently working at Halton Healthcare Services in their breastfeeding clinic at their Oakville location.
